# Evolution of Cluster Morphology and Its Impact on Dislocation Behavior in a Strip-Cast HSLA Steel

**DOI:** 10.3390/ma18245671

**Published:** 2025-12-17

**Authors:** Huiwen Yu, Yuhe Huang, Jun Lu, Junheng Gao, Haitao Zhao, Honghui Wu, Chaolei Zhang, Shuize Wang, Xinping Mao

**Affiliations:** 1 Institute for Carbon Neutrality, University of Science and Technology Beijing, Beijing 100083, China; d202210633@xs.ustb.edu.cn (H.Y.); wuhonghui@ustb.edu.cn (H.W.);; 2 Institute of Steel Sustainable Technology, Liaoning Academy of Materials, Shenyang 110000, China

**Keywords:** high-strength low-alloy steel, strip casting, cluster, dislocation

## Abstract

Strip casting presents a sustainable route for producing advanced steels, such as high-strength low-alloy (HSLA) grades. This study investigated how early-stage isothermal holding (120–1800 s at 923 K) affects the evolution of cluster morphology and its subsequent impact on dislocation behavior and mechanical properties in a strip-cast Nb-bearing HSLA steel. Advanced characterization (atom probe tomography) revealed that prolonged holding promotes the growth of nanoscale Nb-(C,N) clusters and precipitates, accompanied by an increase in ferrite fraction. Remarkably, this evolution simultaneously enhances both strength and ductility. Enhanced ductility and sustained work hardening are linked to a higher density and volume fraction of nanoscale particles, which act as potent obstacles for dislocation nucleation and multiplication. These findings establish a critical link between cluster evolution and dislocation-mediated strengthening, providing a basis for optimizing strip-cast steels.

## 1. Introduction

In recent years, strip casting has emerged as a promising approach in the field of steel production owing to its ability to reduce energy use and greenhouse gas emissions. By combining solidification and hot rolling in a compact line (~50 m), this near-net-shape process eliminates reheating and utilizes rapid solidification rates (10^2^–10^3^ °C/s) to produce thin strips directly, followed by a single hot-rolling pass [1,2,3,4]. Compared with conventional continuous casting, strip casting markedly lowers energy consumption and the carbon footprint, making it attractive for advanced green steels [5].

High-strength low-alloy (HSLA) steels are particularly suited for strip casting due to their mechanical performance and cost-effective microalloying with Nb, V, and Ti [6,7,8]. Their strength mainly arises from nanoscale carbonitride precipitates [9,10]. Wang et al. [11] reported that nanoscale clusters evolve from embryonic states into Guinier–Preston (GP)-like monolayers, and then into precipitates. Similar sequential cluster-to-precipitate transformations and coarsening were later confirmed in Ti–Mo-bearing steels under isothermal conditions [12,13,14]. The advent of atom probe tomography (APT) has further highlighted the role of solute clusters—atomic-scale aggregates without long-range order—as effective strengtheners, analogous to cluster hardening in Al–Cu alloys [15]. This mechanism provides a pathway to achieve both strength and ductility in steels.

The unique rapid solidification of strip casting produces a supersaturated matrix, which upon aging or isothermal holding promotes cluster and fine precipitate formation [1,16]. Aging has been shown to markedly increase strength through cluster formation [1,17]. However, previous studies have only examined the impact of such clusters on the strength–ductility balance, without addressing how these clusters formed during heat treatment, influence dislocation behavior, and contribute to strengthening and ductilizing. Moreover, variations in the coiling process revealed morphological transitions from embryonic monolayer clusters to multilayered clusters [18], which directly affect dislocation multiplication and the balance of strength and ductility. Nevertheless, the evolution of clusters during the critical early stages of isothermal holding and their interactions with dislocations remain insufficiently understood.

To address this, we systematically investigated nanoscale cluster evolution and its impact on dislocation behavior in strip-cast HSLA steel during early-stage isothermal holding. By combining advanced characterization of clusters, precipitates, and dislocations, we clarify how nanoscale transformations govern mechanical response. These insights provide a basis for optimizing the properties of sustainable strip-cast HSLAs.

## 2. Experimental Section

A strip-cast Nb-bearing HSLA steel with a composition of Fe–0.025C–1.4Mn–0.4Si–0.2Cr–0.05Nb–0.004N (wt.%) was investigated. Sample bars (50 mm × 15 mm × 1.2 mm) were austenitized at 1473 K for 300 s, then isothermally held at 923 K for 120, 300, and 1800 s in a salt bath, followed by water quenching. Microstructure was characterized using optical microscopy (Zeiss AxioVert A1, Carl Zeiss, Oberkochen, Germany), scanning electron microscopy (TESCAN MIRA4, Brno, Czech Republic) with Electron Backscatter Diffraction (EBSD, Oxford Symmetry S2, UK, step size 0.1 µm), and transmission electron microscopy (TEM, TECNAI G2 20 at 200 kV, Hillsboro, OR, USA). Atom probe tomography (APT) specimens were prepared via Focused Ion Beam (FIB, Helios G4 CX, Thermo Fisher Scientific, Hillsboro, OR, USA) and analyzed on a LEAP 6000XR (CAMECA Instruments, Madison, WI, USA). Due to the more pronounced artifact in APT analysis for C compared to substitutional elements [19,20], Nb peaks and molecular NbN ions peaks in the APT spectrum were used to represent solute atoms for analysis. Based on the nearest neighbor distribution, the parameter d_max_, the distance between solute atoms was 1 nm, and clusters containing less than 6 solute atoms (N_min_) were ignored. The volume fraction and number density of these particles were estimated from APT results. The former was calculated by dividing the number of atoms within particles by the total number of atoms [21], and the latter was determined by dividing the particle count by the total volume of the needle-shaped sample [20]. Tensile tests were performed on an MTS E45.305 machine (MTS Systems Corporation, Eden Prairie, MN, USA) at a strain rate of 1 mm/min. Dislocation density was measured using an X-ray diffractometer (D8 ADVANCE XRD, Bruker AXS, Karlsruhe, Germany) with a Cu target. Diffraction angles (2θ) ranging from 40° to 120° were measured with a step size of 0.02° under a voltage of 40 kV and a current of 40 mA. The dislocation density was calculated using the Williamson–Hall (WH) equation [22] by analyzing five bcc diffraction peaks ((110), (200), (211), (220), and (310)) planes to calculate the microstrain (e) first, and then computed by the following equation, $\rho =14.4e^{2}/b^{2}$ [23].

## 3. Results and Discussion

**Figure 1:**
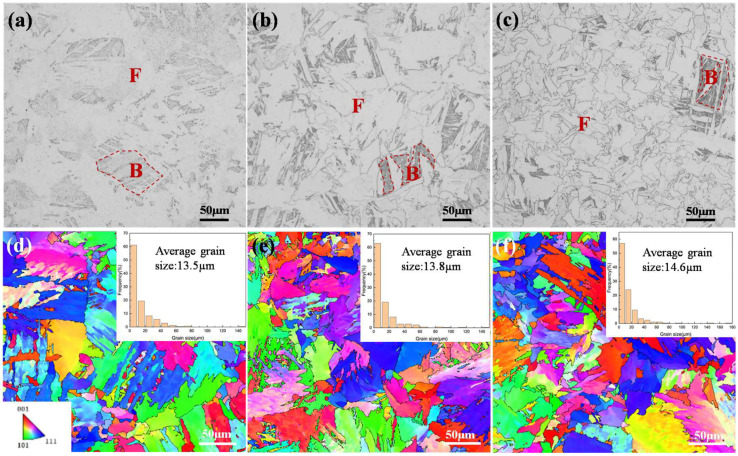
Evolution of microstructure with isothermal holding time at 923 K. (**a**–**c**) Optical micrographs showing polygonal ferrite (F) and bainite (B). (**d**–**f**) EBSD-derived orientation maps and grain size distributions for holding periods of 120 s, 300 s, and 1800 s, respectively.

Figure 1 shows the microstructures of samples subjected to isothermal holding at 923 K for 120 s, 300 s, and 1800 s, respectively. These microstructures consist of polygonal ferrite (F) and bainite (B) with the ferrite volume fraction progressively increasing from 64% to 74%, and then to 82% as the holding time increased. The average grain sizes measured via EBSD were 13.5 µm, 13.8 µm, and 14.6 µm for the respective holding times.

**Figure 2:**
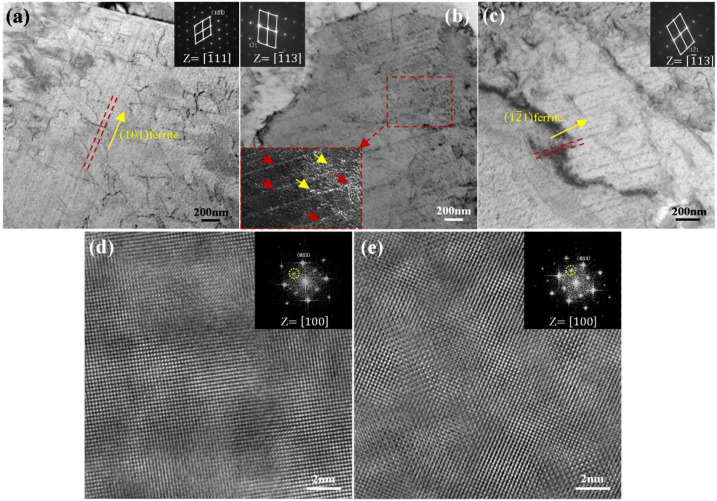
TEM characterization of samples. (**a**–**c**) TEM images revealing interphase precipitation within ferrite grains for holding periods of 120 s, 300 s, and 1800 s, respectively. (**d**,**e**) HRTEM images of samples isothermally held for 120 s and 1800 s, respectively.

Figure 2 presents TEM images of 120 s, 300 s, and 1800 s samples and high-resolution TEM (HRTEM) images of 120 s and 1800 s samples. TEM identified finely dispersed, row-like precipitates formed during the γ→α transformation [24,25] exhibiting irregular spacing and random alignment (Figure 2b). The selected area diffraction pattern (SADP) indicates that precipitate rows shifted from nearly parallel to (101)α (120 s) toward (121)α (1800 s). Such planes generally correspond to coherent or semi-coherent ferrite interfaces like (110)α [24,25,26,27], although incoherent variants may also appear. Similar interfacial precipitation orientations, e.g., (110)α and near {112}α, have been reported in Ti–Mo steels [26], highlighting the generality of this behavior. HRTEM images of 120 s and 1800 s samples (Figure 2d,e) were taken along the [100]α zone axis. The coherent nature of the particles resulted in their weak visibility under standard TEM imaging.

**Figure 3:**
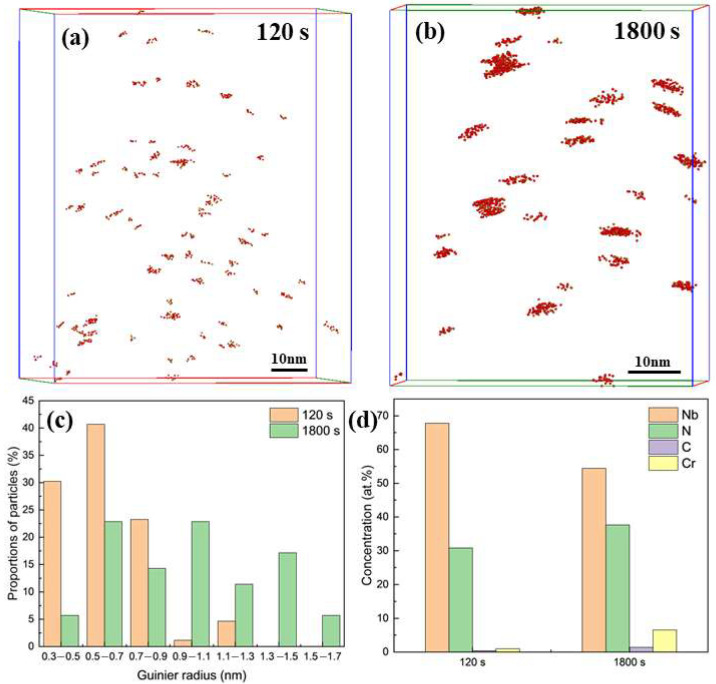
Nanoscale characterization of particles. (**a**,**b**) Three-dimensional atom map reconstructions from APT analysis of samples isothermally held for 120 s and 1800 s, respectively. (**c**) Size distribution of particles expressed by Guinier radius (r_G_). (**d**) Chemical composition of particles.

Therefore, to quantitatively analyze the particle size and composition, atom probe tomography (APT) data were processed using the maximum separation method of the 120 s and 1800 s samples, as illustrated in Figure 3a,b. The size distribution of particles, expressed in terms of the Guinier radius (r_G_), is summarized in Figure 3c. Guinier radii, r_G_, were calculated from the data of gyration radii (I_g_) used by equation $r_{G}=\sqrt{5/3}I_{g}$. In the 120 s sample, 94% of particles had an r_G_ smaller than 0.9 nm, with an average r_G_ of 0.62 nm. These particles were predominantly irregular in shape. By contrast, after 1800 s of holding, only 43% of particles remained below 0.9 nm, and the average r_G_ increased to 0.98 nm. These particles coarsened into disc-like morphologies, tending toward a more spherical shape as they grew, a phenomenon consistent with previous reports in Nb-bearing steels. APT quantification further showed that the volume fraction of particles increased from 0.0033% to 0.0155%, while the number density slightly decreased from 1.88 × 10^23^ m^−3^ to 1.82 × 10^23^ m^−3^ (as shown in Table 1). This trend suggests that these particles grew at the expense of smaller neighboring clusters with fewer atoms. In addition, compositional analysis confirmed that these particles were enriched in Nb and N, along with minor amounts of C and Cr. Notably, the 1800 s sample contained a higher proportion of carbon, and the atomic ratio of Nb to (N+C) approached unity compared to the 120 s sample, suggesting the formation of more stoichiometric carbonitrides with prolonged aging.

APT analyses indicate that initial Nb–N clusters formed as monolayers on {100} matrix planes [17], acting as embryonic precursors that evolved into multilayered clusters, analogous to Guinier–Preston (GP) zones in Al–Cu alloys [28]. This sequential transformation—from monolayer clusters to multilayered clusters and eventually stable precipitates—was driven by Nb segregation at the migrating γ/α interface, where solute drag slows boundary migration, concentrating Nb, N, and C, facilitating cluster decomposition and the nucleation of larger precipitates [29]. Continuous γ/α interface advancement resulted in periodic interphase precipitation.

**Figure 4:**
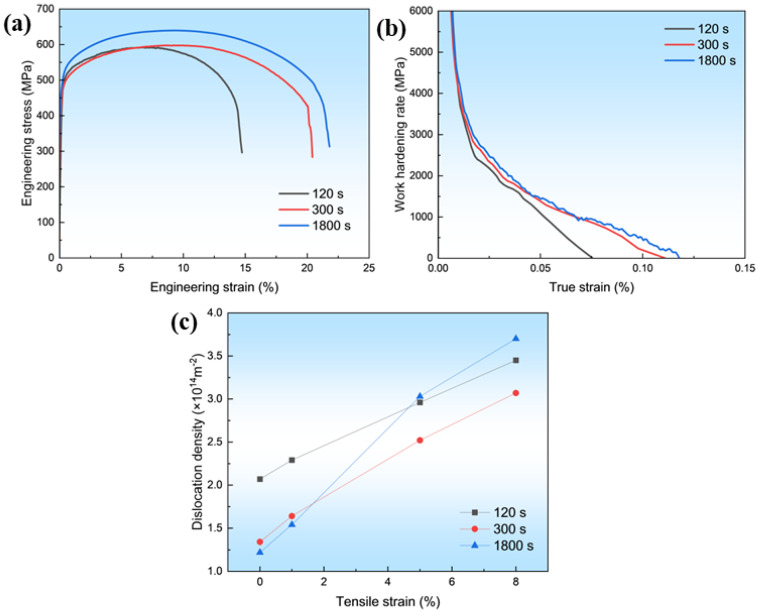
Correlation of mechanical response with deformation microstructure. (**a**) Tensile behavior. (**b**) Work hardening rates. (**c**) Evolution of dislocation density with strain.

To understand the mechanical property variation of specimens with different isothermal holding times, uniaxial tensile tests at room temperature were performed. Tensile tests (Figure 4a) showed that both strength and elongation increased with holding time from 120 s to 1800 s, reflecting hardening from the evolving nanoscale particles. Work hardening rates (Figure 4b) initially declined rapidly, and then decreased gradually; the 120 s sample showed the highest initial rate, whereas the 1800 s sample maintained superior hardening at larger strains. XRD revealed that dislocation density increased with strain in all samples, but more rapidly in those with longer holding, with the 1800 s sample exhibiting the most pronounced rise (Figure 4c). The higher particle density in the ferrite matrix acted as an effective obstacle to dislocation glide, promoting dislocation multiplication and storage [12], thereby enhancing strain hardening.

**Figure 5:**
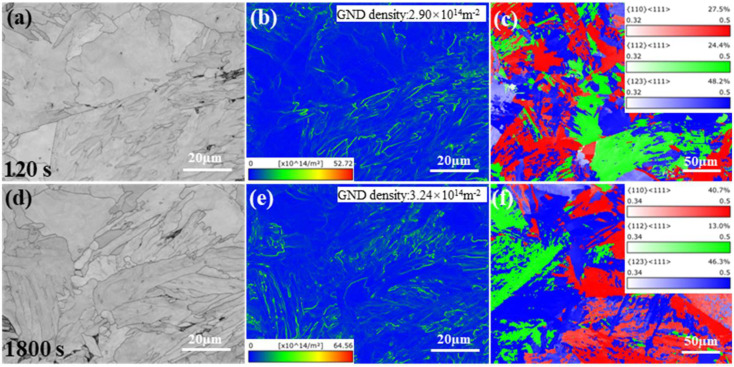
EBSD maps of deformed samples: (**a**,**d**) band contrast, (**b**,**e**) GND density, and (**c**,**f**) Schmid factor maps for 120 s and 1800 s samples interrupted at 8% tensile strain.

Figure 5 presents the EBSD maps of deformed samples. The analysis at 8% strain showed higher geometrically necessary dislocation (GND) density in the 1800 s sample, consistent with stronger strain gradients induced by dense and volume fraction nanoscale particles (Figure 5b,e) [30]. Schmid factor analysis revealed that the 1800 s sample had more grains favorably oriented for {110}<111> slip and fewer for {112}<111>, influencing work hardening through variation in dislocation multiplication and cross-slip capacity [31,32].

**Figure 6:**
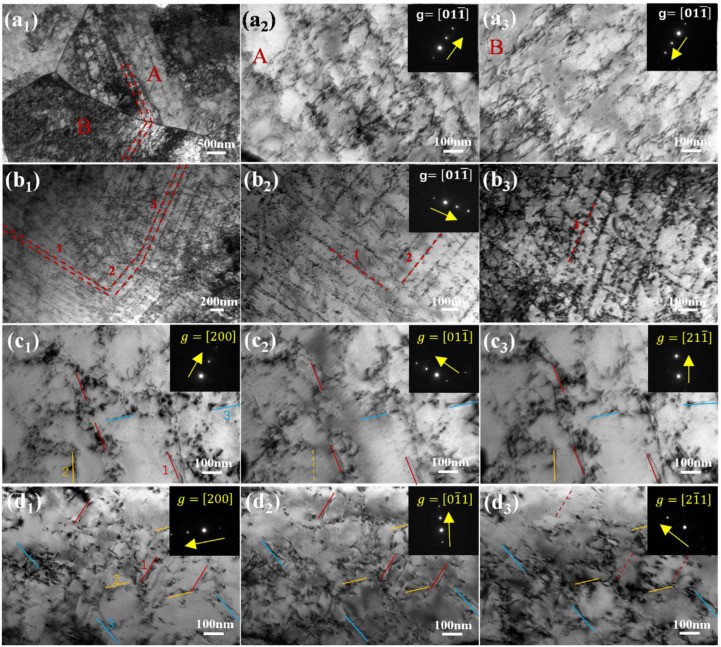
TEM characterization of dislocation structures and their Burgers vector analysis in specimens deformed to 8% strain. General dislocation distributions are shown for (**a1**–**a3**) 120 s and (**b1**–**b3**) 1800 s samples. Corresponding two-beam TEM images near the [011]α zone axis for (**c1**–**c3**) 120 s and (**d1**–**d3**) 1800 s samples were used to determine dislocation visibility (solid lines: visible; dashed lines: invisible) under different g vectors for Burgers vector analysis.

TEM observations of samples deformed to 8% strain (Figure 6) revealed that in the 120 s sample, particles aligned in rows within grains, inducing dislocation loops and pinning points. In the 1800 s sample, high particle density and volume fraction with multidirectional arrangements promoted more homogeneous dislocation distribution and extensive looping. Burgers vector (b) analysis using the g·b = 0 criterion confirmed both screw and mixed 1/2<111> dislocations (as shown in Table 2). The presence of screw dislocations capable of cross-slip facilitates dislocation multiplication and entanglement around particles, contributing to the sustained work hardening observed in the 1800 s sample.

The superior mechanical properties and sustained work hardening of the 1800 s sample are attributed to the growth, increased volume fraction, and high density of nanoscale particles (r_G_: 0.62 → 0.98 nm), which effectively impede dislocation glide. This promotes extensive dislocation multiplication, activation of multiple slip systems—particularly {110}<111>—and cross-slip of screw dislocations, leading to complex dislocation networks pinned by particles. Consequently, both GNDs and statistically stored dislocations (SSDs) were retained, explaining the enhanced work hardening at larger strains.

## 4. Conclusions

The evolution of nanoscale Nb–N clusters into stable carbonitrides during isothermal holding was shown to strongly influence the deformation behavior of strip-cast HSLA steel. Particle growth and increased volume fraction effectively promoted dislocation multiplication and cross-slip, leading to enhanced strain hardening. As a result, both strength and ductility improved with prolonged holding, underscoring the importance of controlling cluster–precipitate transformation for optimizing the mechanical performance of strip-cast green steels.

## Figures and Tables

**Table 1:** Number density, volume fraction, and average Guinier radius of particles.

Sample	Number Density/×10^23^ m^−3^	Volume Fraction/%	Average Guinier Radius/nm
120 s	1.88	0.0033	0.62
1800 s	1.82	0.0155	0.98

**Table 2:** Contrast of dislocations in different operation reflections; “√” represents visible and “×” invisible.

Sample	$g=\left[200\right]$	$g=\left[01\bar{1}\right]$	$g=\left[21\bar{1}\right]$	$g=\left[0\bar{1}1\right]$	$g=\left[2\bar{1}1\right]$	b	Dislocation Type
120s-1	√	√	√			$\left[11\bar{1}\right]$	Screw
120s-2	√	×	√			$\left[111\right]/\left[\bar{1}11\right]$	Mixed
120s-3	√	√	√			$\left[11\bar{1}\right]$	Mixed
1800s-1	√			√	×	$\left[11\bar{1}\right]$	Screw
1800s-2	√			√	√	$\left[1\bar{1}1\right]$	Mixed
1800s-3	√			√	√	$\left[1\bar{1}1\right]$	Screw

## Data Availability

Original contributions presented in this study are included in the article. Further inquiries can be directed to the corresponding authors.
